# Supplementary material for: Germline Variation in PDCD1 Is Associated with Overall Survival in Patients with Metastatic Melanoma Treated with Anti-PD-1 Monotherapy
Source: Cancers (Basel). 2021 Mar 18;13(6):1370. doi: 10.3390/cancers13061370 (PMC8002987; doi:10.3390/cancers13061370)
Supplement: Supplementary file 1 [file cancers-13-01370-s001.pdf]

Supplementary Material: Germline Variation in PDCD1 Is Associated with Overall Survival in Patients with Metastatic Melanoma Treated with Anti-PD-1 Monotherapy

Mirjam de With, Daan P. Hurkmans, Esther Oomen-de Hoop, Ayoub Lalouti, Sander Bins, Samira El Bouazzaoui, Mandy van Brakel, Reno Debets, Joachim G. J. V. Aerts, Ron H. N. van Schaik, Ron H. J. Mathijssen and Astrid A. M. van der Veldt

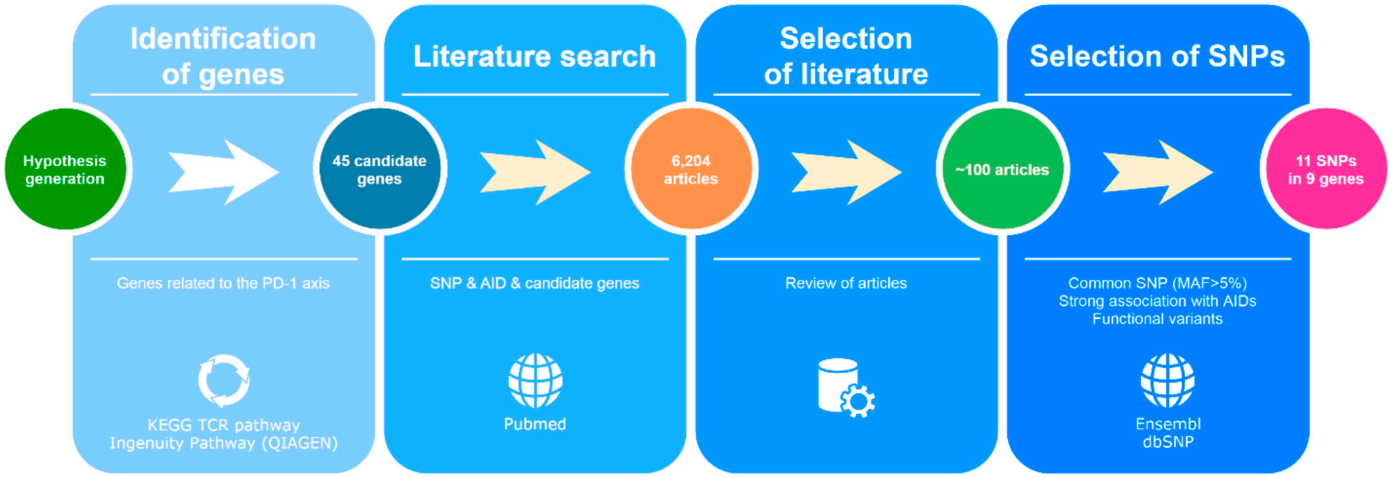

Figure S1. Search strategy for the selection of single-nucleotide polymorphisms (SNPs). Abbreviations: PD-1: programmed cell death-1; KEGG TCR: Kyoto Encyclopedia of Genes and Genomes T cell receptor; SNP: single-nucleotide polymorphism; AID: autoimmune disease; MAF: minor allele frequency.

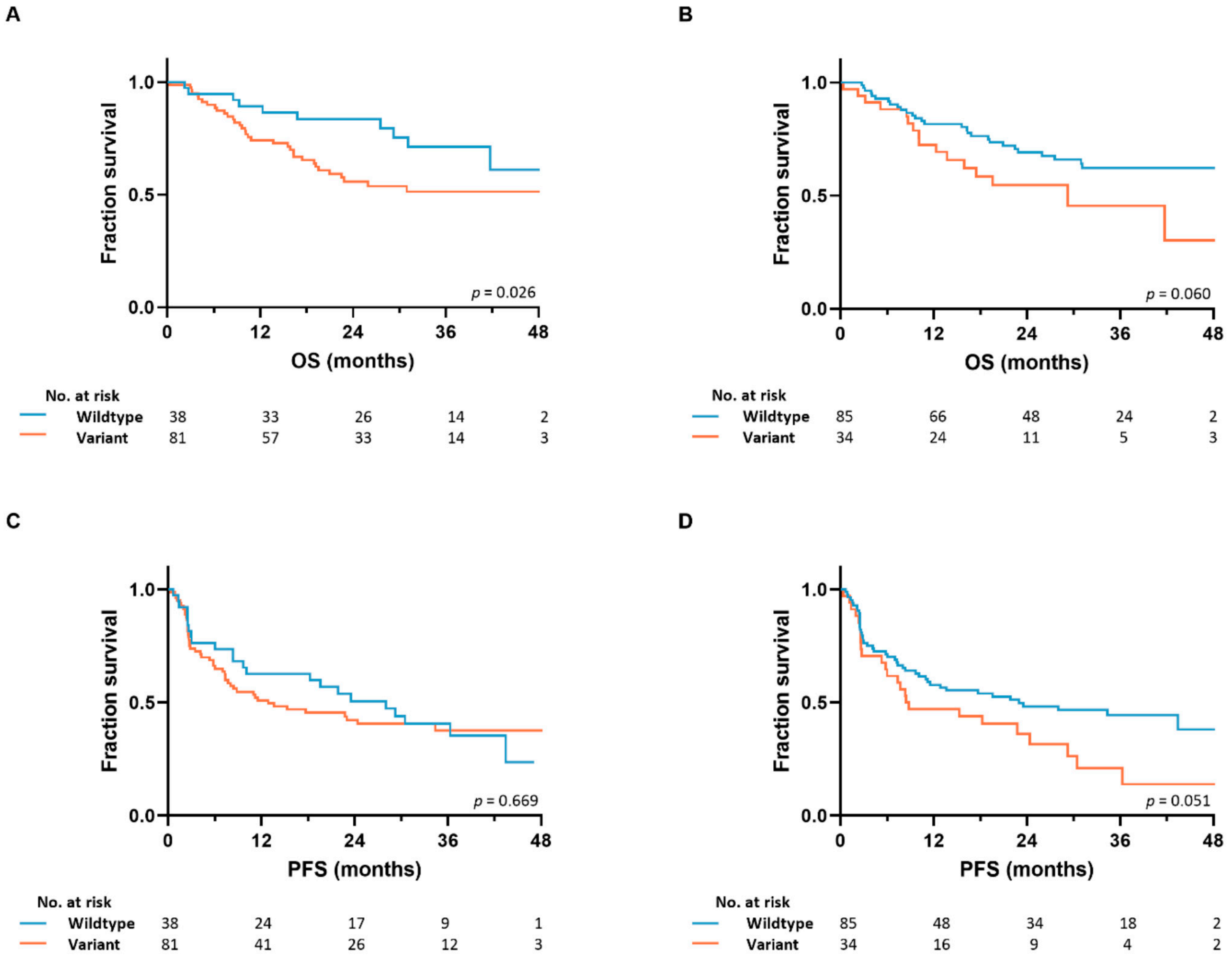

Figure S2. Kaplan-Meier plots showing the overall survival (OS) and progression free survival (PFS) of patients included in this study (N = 119) according to PDCD1 804C>T (**A**,**C**) and GZMB c.128C>A (**B**,**D**). In blue the wild type patients; in orange the variant homozygous and heterozygous allele carriers.

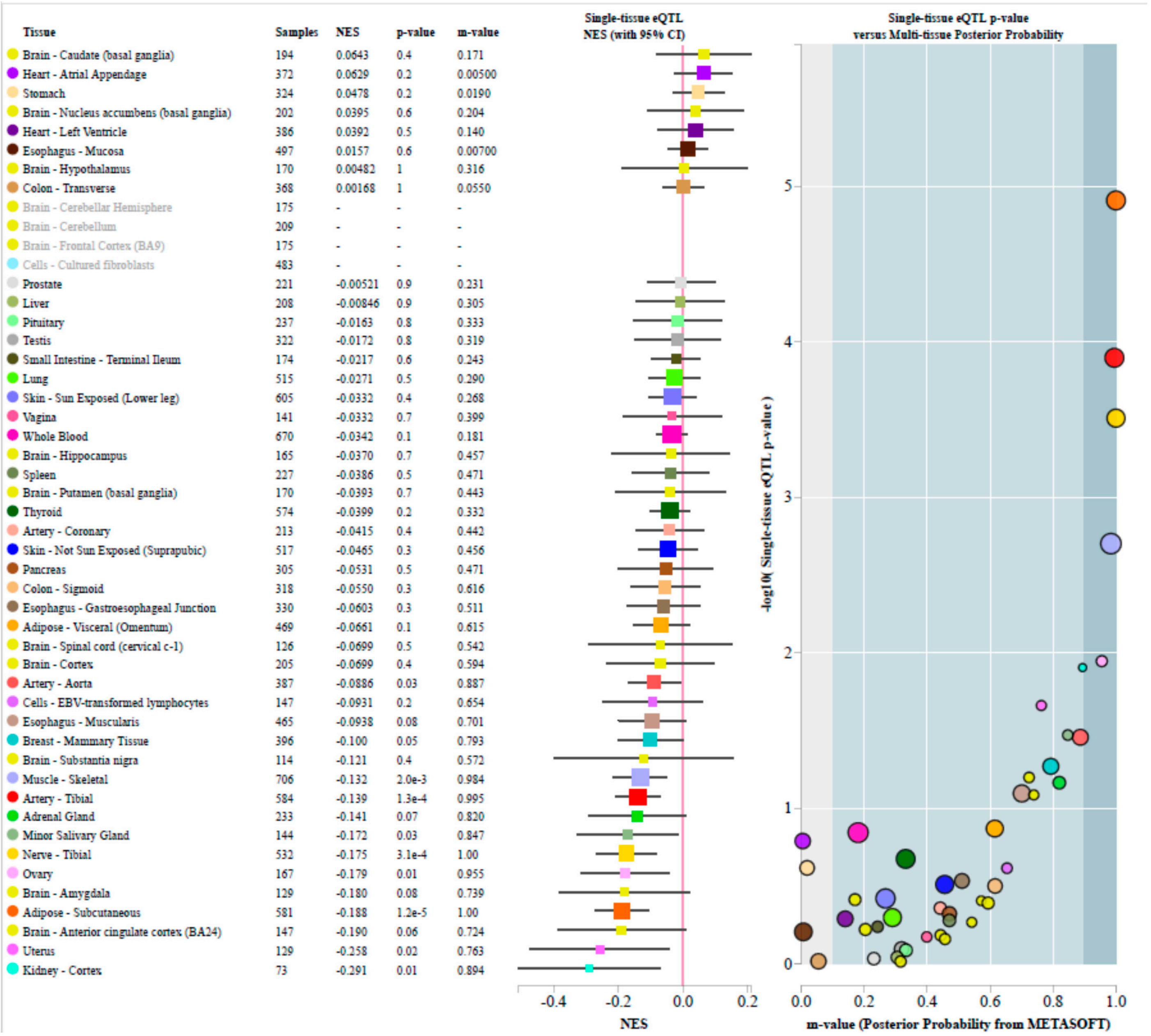

Tissue	Samples	NES	p-value	m-value
Brain - Caudate (basal ganglia)	194	0.0643	0.4	0.171
Heart - Atrial Appendage	372	0.0629	0.2	0.00500
Stomach	324	0.0478	0.2	0.0190
Brain - Nucleus accumbens (basal ganglia)	202	0.0395	0.6	0.204
Heart - Left Ventricle	386	0.0392	0.5	0.140
Esophagus - Mucosa	497	0.0157	0.6	0.00700
Brain - Hypothalamus	170	0.00482	1	0.316
Colon - Transverse	368	0.00168	1	0.0550
Brain - Cerebellar Hemisphere	175	-	-	-
Brain - Cerebellum	209	-	-	-
Brain - Frontal Cortex (BA9)	175	-	-	-
Cells - Cultured fibroblasts	483	-	-	-
Prostate	221	-0.00521	0.9	0.231
Liver	208	-0.00846	0.9	0.305
Pituitary	237	-0.0163	0.8	0.333
Testis	322	-0.0172	0.8	0.319
Small Intestine - Terminal Ileum	174	-0.0217	0.6	0.243
Lung	515	-0.0271	0.5	0.290
Skin - Sun Exposed (Lower leg)	605	-0.0332	0.4	0.268
Vagina	141	-0.0332	0.7	0.399
Whole Blood	670	-0.0342	0.1	0.181
Brain - Hippocampus	165	-0.0370	0.7	0.457
Spleen	227	-0.0386	0.5	0.471
Brain - Putamen (basal ganglia)	170	-0.0393	0.7	0.443
Thyroid	574	-0.0399	0.2	0.332
Artery - Coronary	213	-0.0415	0.4	0.442
Skin - Not Sun Exposed (Suprapubic)	517	-0.0465	0.3	0.456
Pancreas	305	-0.0531	0.5	0.471
Colon - Sigmoid	318	-0.0550	0.3	0.616
Esophagus - Gastroesophageal Junction	330	-0.0603	0.3	0.511
Adipose - Visceral (Omentum)	469	-0.0661	0.1	0.615
Brain - Spinal cord (cervical c-1)	126	-0.0699	0.5	0.542
Brain - Cortex	205	-0.0699	0.4	0.594
Artery - Aorta	387	-0.0886	0.03	0.887
Cells - EBV-transformed lymphocytes	147	-0.0931	0.2	0.654
Esophagus - Muscularis	465	-0.0938	0.08	0.701
Breast - Mammary Tissue	396	-0.100	0.05	0.793
Brain - Substantia nigra	114	-0.121	0.4	0.572
Muscle - Skeletal	706	-0.132	2.0e-3	0.984
Artery - Tibial	584	-0.139	1.3e-4	0.995
Adrenal Gland	233	-0.141	0.07	0.820
Minor Salivary Gland	144	-0.172	0.03	0.847
Nerve - Tibial	532	-0.175	3.1e-4	1.00
Ovary	167	-0.179	0.01	0.955
Brain - Amygdala	129	-0.180	0.08	0.739
Adipose - Subcutaneous	581	-0.188	1.2e-5	1.00
Brain - Anterior cingulate cortex (BA24)	147	-0.190	0.06	0.724
Uterus	129	-0.258	0.02	0.763
Kidney - Cortex	73	-0.291	0.01	0.894

Figure S3. Multi-tissue expression quantitative trait loci (eQTL) analysis, showing separate eQTL statistics for germline variation PDCD1 804C > T. Global RNA expression levels in tissues from different organ sites in the Genotype-Tissue Expression (GTEx) database (https://gtexportal.org, accessed on 17 May 2020) were analyzed as quantitative traits (left side of this figure). Variations in gene expression that are highly correlated with genetic variations can be identified as eQTLs. The normalized effect size (NES) with the 95% confidence interval (95%CI) of the eQTLs is defined as the slope of the linear regression, and is computed as the effect of the minor allele T to major allele C. A negative NES indicates lower gene expression of PDCD1 for the minor allele T. *p*-values < 0.05 were considered siginifcant. Size of dots and squares (right side of this figure) are correlated with the number of samples included in the analysis. The figure was created in the GTEx portal (https://gtexportal.org).

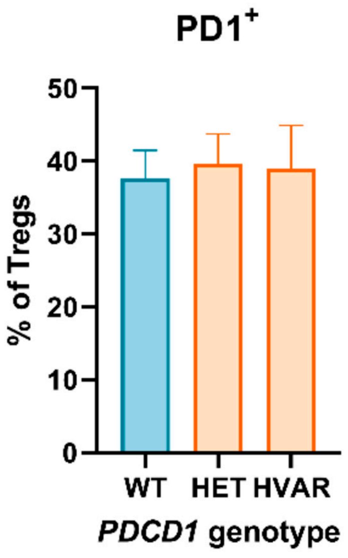

Figure S4. PD-1 expression in peripheral regulatory T cells according to PDCD1 804C > T genotypes (comparing wild type [WT], heterozygous variant [HET] and homozygous variant [HVAR]). Peripheral T cells were collected prior to anti-PD-1 monotherapy in patients with metastatic melanoma (*N* = 47).

Table S1. Univariable associations of clinical baseline characteristics and SNPs with PFS.

	Test Variables	HR (95% CI)	*p* Value	Bias Corrected 95% CI
**Clinical baseline characteristics**				
ECOG PS	1 + 2 vs. 0	2.132 (1.299–3.499)	0.003	1.327–3.533
LDH	Continue (U/L)	1.002 (1.000–1.003)	0.051	0.999–1.003
Prior treatment	Yes vs. no	1.642 (0.991–2.720)	0.054	0.938–2.732
BRAF V600E/K mutation	Present vs. absent	1.595 (0.986–2.581)	0.057	1.016–2.566
CNS metastasis	Yes vs. no	1.523 (0.759–3.057)	0.237	
Gender	Female vs. male	0.913 (0.557–1.496)	0.718	
Age	≥ 65 years vs. < 65 years	0.946 (0.588–1.521)	0.818	
**Variants: dominant model**				
GZMB c.128C > A	CA + AA vs. CC	1.633 (0.999–2.670)	0.051	1.030–2.561
IL10 c.378 + 284G > T	GT + TT vs. GG	0.697 (0.403–1.203)	0.195	
PTPN11 333-233A > G	AG + GG vs. AA	1.402 (0.791–2.483)	0.247	
IFNG -1616T > C	TC + CC vs. TT	1.124 (0.701–1.800)	0.628	
IL2RA c.64 + 5006A > G	AG + GG vs. AA	1.116 (0.687–1.813)	0.657	
PDCD1 804C > T	CT + TT vs. CC	1.115 (0.676–1.838)	0.669	
IFNG 367-895C > T	CT + TT vs. CC	0.933 (0.579–1.504)	0.777	
IL2RB -34 + 1055G > A	GA + AA vs. GG	0.969 (0.605–1.553)	0.896	
ZAP70 -21-4127C > A	CA + AA vs. CC	1.022 (0.639–1.635)	0.928	
HLAA c.del/TTTA	del + del/TTTA vs. insTTTA	0.986 (0.615–1.582)	0.955	
IFNG c.874A > T	AT + TT vs. AA	1.002 (0.573–1.752)	0.994	
**Variants: recessive model**				
IL2RA c.64 + 5006A > G	GG vs. AA + AG	1.563 (0.893–2.737)	0.118	
IFNG -1616T > C	CC vs. TT + TC	0.663 (0.303–1.451)	0.303	
IFNG 367-895C > T	TT vs. CC + CT	1.196 (0.664–2.153)	0.551	
IFNG c.874A > T	TT vs. AA + AT	0.940 (0.564–1.568)	0.813	
PDCD1 804C > T	TT vs. CC + CT	1.065 (0.609–1.862)	0.825	

Univariable associations of clinical baseline characteristics and single-nucleotide polymorphisms (SNPs) with progression free survival (PFS) (N = 119). Abbreviations: CI: Confidence Interval; ECOG PS: Eastern Cooperative Oncology Group Performance Status; LDH: Serum lactate dehydrogenase; CNS: central nervous system; Del: deletion.

Table S2. Univariable associations of clinical baseline characteristics and SNPs with BOR.

	Test Variables	OR (95% CI)	*p* value	Bias corrected 95% CI
**Clinical baseline characteristics**				
ECOG PS	1 + 2 vs. 0	0.452 (0.185–1.104)	0.082	0.179–1.140
Prior treatment	Yes vs. no	0.481 (0.196–1.185)	0.112	
BRAF V600E/K mutation	Present vs. absent	0.526 (0.226–1.224)	0.136	
CNS metastasis	Yes vs. no	0.420 (0.128–1.383)	0.154	
Age	≥ 65 years vs. < 65 years	1.826 (0.770–4.333)	0.172	
LDH	Continue (U/L)	0.999 (0.996–1.002)	0.628	
Gender	Female vs. male	1.077 (0.458–2.536)	0.864	
**Variants: dominant model**				
HLAA c.del/TTTA	del + del/TTTA vs. insTTTA	0.565 (0.247–1.293)	0.177	
IL2RB -34 + 1055G > A	GA + AA vs. GG	1.481 (0.650–3.379)	0.350	
IFNG -1616T > C	TC + CC vs. TT	0.689 (0.301–1.580)	0.379	
PTPN11 333-233A > G	AG + GG vs. AA	0.667 (0.241–1.845)	0.435	
IL2RA c.64 + 5006A > G	AG + GG vs. AA	1.390 (0.605–3.192)	0.438	
ZAP70 -21-4127C > A	CA + AA vs. CC	1.377 (0.596–3.182)	0.454	
IFNG 367-895C > T	CT + TT vs. CC	0.728 (0.311–1.705)	0.465	
IL10 c.378 + 284G > T	GT + TT vs. GG	1.388 (0.552-3.491)	0.486	
IFNG c.874A > T	AT + TT vs. AA	0.698 (0.253–1.925)	0.487	
GZMB c.128C > A	CA + AA vs. CC	1.178 (0.465–2.985)	0.729	
PDCD1 804C > T	CT + TT vs. CC	0.986 (0.410–2.373)	0.976	
**SNPs: recessive model**				
IFNG 367-895C > T	TT vs. CC + CT	0.667 (0.241–1.845)	0.435	
IL2RA c.64 + 5006A > G	GG vs. AA + AG	0.724 (0.264–1.998)	0.531	
PDCD1 804C > T	TT vs. CC + CT	1.346 (0.486–3.725)	0.567	
IFNG -1616T > C	CC vs. TT + TC	1.369 (0.355–5.272)	0.648	
IFNG c.874A > T	TT vs. AA + AT	0.910 (0.377–2.199)	0.834	

Univariable associations of clinical baseline characteristics and single-nucleotide polymorphisms (SNPs) with best overall response (BOR) (n = 117). Abbreviations: OR: Odds Ratio; CI: Confidence Interval; ECOG PS: Eastern Cooperative Oncology Group Performance Status; CNS: central nervous system; LDH: Serum lactate dehydrogenase; Del: deletion.

Table S3. Correlation matrix.

	Age	LDH	ECOG PS	Prior treatment	BRAF V600E/K mutation	GZMB c.128C > A
**LDH**	0.164 $^{\beta}$ p = 0.074					
**ECOG PS**	−0.069 $^{\gamma}$ p = 0.460	0.049 $^{\beta}$ p = 0.603				
**Prior treatment**	−0.121 $^{\gamma}$ p = 0.186	0.094 $^{\beta}$ p = 0.312	0.203 $^{\gamma}$ p = 0.030			
**BRAF V600E/K mutation**	−0.340 $^{\gamma}$ p = <0.001	−0.015 p = 0.873	0.043 $^{\gamma}$ p = 0.644	0.160 $^{\gamma}$ p = 0.080		
**GZMB c.128C > A**	0.155 $^{\gamma}$ p = 0.090	0.002 $^{\beta}$ p = 0.986	0.161 $^{\gamma}$ p = 0.086	0.147 $^{\gamma}$ p = 0.109	−0.096 $^{\gamma}$ p = 0.297	
**PDCD1 804C > T**	0.095 $^{\gamma}$ p = 0.302	−0.051 $^{\beta}$ p = 0.581	0.069 $^{\gamma}$ p = 0.461	−0.059 $^{\gamma}$ p = 0.520	−0.123 $^{\gamma}$ p = 0.179	0.034 $^{\gamma}$ p = 0.709

The correlation matrix is shown of patient characteristics and single-nucleotide polymorphisms (SNPs) with $p < 0.10$ in univariate OS or PFS analysis (N = 119). Abbreviations: LDH: Serum lactate dehydrogenase; ECOG: Eastern Cooperative Oncology Group Performance Status. Significant associations ($p < 0.05$) in orange. $^{\beta}$ Rank-biserial correlation coefficient ; $^{\gamma}$ Phi correlation coefficient.
